# Nationwide Lockdown, Population Density, and Financial Distress Brings Inadequacy to Manage COVID-19: Leading the Services Sector into the Trajectory of Global Depression

**DOI:** 10.3390/healthcare9020220

**Published:** 2021-02-17

**Authors:** Donglei Yu, Muhammad Khalid Anser, Michael Yao-Ping Peng, Abdelmohsen A. Nassani, Sameh E. Askar, Khalid Zaman, Abdul Rashid Abdul Aziz, Muhammad Moinuddin Qazi Abro, Sasmoko, Mohd Khata Jabor

**Affiliations:** 1 School of Political Science and Public Administration, Wuhan University, Wuhan 430000, China; yudonglei@163.com; 2 School of Public Administration, Xi’an University of Architecture and Technology, Xi’an 710000, China; 3 School of Economics & Management, Foshan University, Foshan 528000, China; s91370001@mail2000.com.tw; 4 Department of Management, College of Business Administration, King Saud University, Riyadh 11587, Saudi Arabia; Nassani@ksu.edu.sa (A.A.N.); qaziabro@gmail.com (M.M.Q.A.); 5 Department of Statistics and Operations Research, College of Science, King Saud University, Riyadh 11587, Saudi Arabia; saskar@ksu.edu.sa; 6 Department of Economics, University of Haripur, Haripur 22620, Pakistan; khalid.zaman@uoh.edu.pk; 7 Faculty of Leadership and Management, University Sains Islam Malaysia, Nilai 71800, Negeri Sembilan, Malaysia; rashid@usim.edu.my; 8 Primary Teacher Education Department, Faculty of Humanities, Bina Nusantara University, Jakarta 11480, Indonesia; sasmoko@binus.edu; 9 Faculty of Social Sciences & Humanities, Universiti Teknologi Malaysia (UTM), Johor 81310, Malaysia; mkhata@utm.my

**Keywords:** COVID-19, services value-added, lockdown, word-of-mouth, social distancing, quantile regression

## Abstract

The service industry provides distributive services, producer services, personal services, and social services. These services largely breakdowns due to restrictions on border movements, confined travel and transportation services, a decline in international tourists’ visitation, nationwide lockdowns, and maintaining social distancing in the population. Although these measures are highly needed to contain coronavirus, it decreases economic and financial activities in a country, which requires smart solutions to globally subsidize the services sector. The study used different COVID-19 measures, and its resulting impact on the services industry by using world aggregated data from 1975 through 2020. The study benefited from the Keynesian theory of aggregate demand that remains provided a solution to minimize economic shocks through stringent or liberalizing economic policies. The COVID-19 pandemic is more severe than the financial shocks of 2018 that affected almost all sectors of the globalized world, particularly the services sector, which has been severally affected by COVID-19; it is a high time to revisit economic policies to control pandemic recession. The study used quantiles regression and innovation accounting matrix to obtain ex-ante and ex-post analysis. The quantile regression estimates show that causes of death by communicable diseases, including COVID-19, mainly decline the share of services value added to the global GDP at different quantiles distribution. In contrast, word-of-mouth helps to prevent it from the transmission channel of coronavirus plague through information sharing among the general masses. The control of food prices and managing physical distancing reduces suspected coronavirus cases; however, it negatively affects the services sector’s value share. The smart lockdown and sound economic activities do not decrease coronavirus cases, while they support increasing the percentage of the services sector to the global GDP. The innovation accounting matrix suggested that smart lockdown, managing physical distancing, effective price control, and sound financial activities will help to reduce coronavirus cases that will further translate into increased services value-added for the next ten years. The social distancing will exert a more considerable variance error shock to the services industry, which indicates the viability of these measures to contained novel coronavirus over a time horizon. The study used the number of proxies to the COVID-19 measures on the service sector that can be continued with real-time variables to obtain more inferences.

## 1. Introduction

The novel coronavirus leads to novel global depression, as it spread at an exponential rate and increases death tolls accordingly. The current comprehensive statistics of coronavirus cases are, to date, at 62,564,449 and death tolls at 1,458,112. The active cases are 17,917,632, and critical severe cases are 105,238. The USA economy mainly suffers from the coronavirus pandemic and it has added 21.7% registered cases globally. India, Brazil, Russia, and France have been added by 15%, 10.5%, 3.58%, and 3.53%, respectively. The share of Europe in registered cases relative to world economy reached 27%, followed by North America, i.e., 25.3%, Asia, i.e., 26.38%, South America, i.e., 17.67%, Africa, i.e., 3.4%, and Oceania, i.e., 0.07% [1].

The effects of coronavirus are not limited to any single sector, affecting the overall global economy. This study focused on the services sector, as it contributes a share of more than 60% of the world GDP. The percentage of the services sector is more significant in high-income countries (i.e., more than 70%), followed by middle-income countries (i.e., more than 50%) and low-income countries (more than 45%). The services sector provides distributive services (that are related to transportation, means of communications, storage, wholesale businesses, retail businesses, hotels, and restaurants businesses), producer services (referred to financial activities), personal services (related to mass entertainment, recreation activities, ownership, and dwelling), and social services (as compared to public administration, defiance, provision of education, health, and other community services) [2].

The impact of coronavirus (COVID-19) on the services industry is evident, mainly because of the high risk associated with spreading coronavirus; thus, the globalized world has already taken several precautionary steps as per WHO available guidelines, among which few of them are presented, as follows, i.e., maintaining physical distancing between the residents. Therefore, all Giga shopping malls should have been closed; complete and partial lockdowns the streets; school/colleges/universities closures; travel and transport restrictions; international flights cancelled; borders closed; banned massive gatherings; and, limited recreational activities and community services. All of these COVID-19 measures directly affect the services value-added, which is essential in given circumstances. Governments put many efforts into contain coronavirus through national and international strategic unified policies and going towards smart lockdowns to keep relaxing the COVID-19 measures where required. In a short period, many scholarly writings were available on the effects of COVID-19 on different sectors of the economy. The study focused on the impacts of COVID-19 measures on the services industry, as it is considered to be a globalized world’s backbone. The study used some different Boolean operators to assess how many scholarly writings have been carried out for COVID-19 and the services industry via using the Google Scholar search engine. The advanced search is used to limit the given words in the ‘title of the article’. Table 1 shows the presented statistics for ready reference.

The given searched results confirmed the need to explore the impacts of COVID-19 measures on the services industry, as minimal work has been carried out in the given sector. The critical literature has been cited to understand the direction of earlier researches on the stated topic; for instance, Gössling et al. [3] discussed the impact of COVID-19 on international tourism. They argued that the recent pandemic affects the massive global population, due to international travel bans, and it is projected that more than one-quarter of international tourism will be affected relative to 2019. The greater need for strategic policies is desirable for the transportation industry to maintain its growth rate with safety measures. Thams et al. [4] found that the COVID-19 epidemic mainly affected the international tourism industry in the form of the suspended tourism value chain, including restrictions on travel and transportation services within and outside the countries, not to allow tourists for visitation, hotels closure, stopped airlines, cruise lines, and retailers’ activities, which substantially decline the country’s revenue streams. The fair chance is to contain coronavirus through unified tourism policies and make them safer and healthier globally. Ruiz Estrada et al. [5] evaluated the impact of COVID-19 on the Chinese economies into four different sectors, including international tourism, air travel industry, foreign trade, and energy markets. They found that, except for energy markets, the rest of the three sectors negatively contribute to the country’s economic growth while increasing the demand for electricity consumption due to high medical services and quarantine usage will likely perform high in stock market indices. Thus, it has ultimately contributed to the country’s economic growth. Foremny et al. [6] surveyed Spain’s sizeable population regarding public health preferences and their willingness to acquire healthcare services. The results show that the low-income group is mostly disturbed by this pandemic, and their mental health is mainly deteriorating due to continuous lockdown. They require healthcare provision and early recovery, so they could reduce their sufferings, both mentally and financially. Goodell [7] discussed the vulnerability of COVID-19 on global economic and financial structure, and argued that, due to the massive increase in healthcare expenditures for identification and prevention of susceptible coronavirus patients, the cost of medical instruments, protective gadgets, and much other healthcare-related infrastructure put enormous pressure on the global economy, which has to be subsidized by the financial sector. However, banking sectors face many challenges regarding the loan disbursement and loan recoveries that put more stress on the other economic areas. Zhao et al. [8] investigated the outbreak of coronavirus pandemic in Wuhan China through the transmission channel of mainly travel by train. The high need for making a ‘command and control travel system’ may limit the outbreak of COVID-19 to the other cities and the world.

Zheng et al. [9] further concluded that private and public transportation provides a transmission carrier of coronavirus pandemics from one city to another. A positive association was found between the different transportation channels and increased COVID-19 cases from Wuhan China to other cities. A negative correlation was found between the more considerable distances between the Wuhan city and the other cities in spreading coronavirus. Public transportation provides a carrier to transmits coronavirus from Wuhan to nearby cities; thus, the Wuhan city’s complete lockdown was enviable in reducing the coronavirus outbreak to the other cities and the world. Basch et al. [10] concluded that social media could significantly minimize the COVID-19 impacts through knowledge sharing and awareness regarding the pandemic to use prevention measures to the contain pandemic. YouTube is considered to be the most inspiring way to share the coronavirus pandemic and improve community health services. Peyrav et al. [11] considered a case of Iran’s economy that has been negatively affected by the coronavirus, and the sizeable number of registered cases is increasing day-by-day. The Iranian government has worked dedicatedly to contain the coronavirus through massive public education programs and electronic awareness campaigns, while, on the other side, the country is conducting research workshops, training, and increasing healthcare budgets to the control the pandemic. The need for word-of-mouth campaigns regarding coronavirus prevention is vital to promote country resilience. Sahu [12] suggested several policy measures to contain the coronavirus among the students and teaching/administrative staff, as high risk is associated with the educational institutes through close contacts. As per WHO guidelines, the closure of all educational institutes is deemed to be desirable for such an indefinite period until the virus can be controlled accordingly. Nevertheless, these positive measures negatively impact the mental health of students and academic staff. Thus, the need for proper counselling, online teaching courses, assessments, and evaluation is highly desirable in improving students and academic staff’s psychological health. Bhalekar [13] argued that the Indian economy was mainly affected by the coronavirus pandemic due to a random lockdown in a country that increases the daily wagers’ miseries, which led to increasing the source of virus in a country. The COVID-19 affects all major sectors of the Indian economy, not limited to the labor market, educational institutes, electronic commerce, and overall economic growth. The high need for strategic thinking, unified global policies, smart lockdowns, emergency relief packages to the poor laborer, and stable financial markets would help control to the coronavirus resourcefully. Zheng [14] suggested the need for psychological treatment of healthcare workers directly exposed to the coronavirus, and they are more likely concerned about their families and friends. This family support for health care workers is desirable for working with sound health. Sanità di Toppi et al. [15] confined their findings on a more important aspect of spreading coronavirus pandemic related to the airborne particulate, providing a channel to carry coronavirus into the human respiratory system. The urgent need is for making sustainable policies to limit particulate matters and limit the virus accordingly. Musselwhite et al. [16] suggested that public transportation could be a carrier of coronavirus spread, as people are either sitting or standing in a closed environment, while coughing, touching, and sneezing may transmit the microorganism from one to others. The doors, ticket machines, windows, elevators, seats, and many other areas could be possible places of the infectious disease. Deng and Peng [17] found that the case-fatality ratio is mainly evident with the following symptoms of coronavirus, including high fever, too much coughing, shortness of breath, and chest pain, while other comorbidities of the fatality cases, including high stress, heart patients, diabetes, cerebral infarction, and chronic bronchitis. These healthcare concerns that are required more good policies to reduce the case fatality ratio, while symptomatic treatment is provided to the coronavirus patients until the possible medication and the vaccine is not invented. Anser et al. [18] considered a panel of 76 countries using time series data from 2010–2019 to evaluate the impact of COVID-19 measures on global poverty, and found that population density, lack of necessary sanitation facilities, environmental challenges, and death by communicable diseases put a significant burden on the low-income group, which could be minimized by increasing public healthcare expenditures across countries. The need for pro-poor growth policies will support the breakdown of the vicious cycle of poverty that would be further translated into sustained economic growth.

The significant discussion that is based on earlier literature emphasized the need to evaluate the possible impacts of COVID-19 measures on services industries while using an aggregated world data level. Fewer studies on the stated topic give room to investigate to select the specified area, which would provide more policy insights for analyzing the services industry’s response against the COVID-19 measures. The objectives of the study are as follows:

(I) To examine the possible impacts of COVID-19 measures on the global services industry.

(II) To investigate the direct effect of communicable diseases, including COVID-19, on services value-added.

(III) To determine the role of word-of-mouth against the coronavirus pandemic and its possible impact on the services industry and

(IV) To observe the effects of lockdown, social distancing, price control, and financial activities on the services industry.

Different countries have widely adopted these measures against the coronavirus that analyzed in the study of the services sector. The study used quantile regression estimates to analyze the predictors’ different variations on the response variable at different quantiles distribution. This technique is better in a given scenario that will provide robust inferences.

## 2. Data Sources and Methodological Framework

There are some COVID-19 measures that have been used to control the pandemic at a global scale, and a few of them are listed below, i.e.,

(I) Information Sharing: the right information with correct facts and figures are the responsibility of every government to share with their residents, while, at an international platform, the WHO and other international agencies have to prepare the policy documents regarding prevention from novel coronavirus and spread it through different information channels. The national and international agencies have already provided the right information through various communication channels, and it is now a duty to respond to the general masses to act like a civilized person. The ‘word-of-mouth’ mostly used the word in marketing the specified products where information is shared from one person to another through oral communication [19]. The adult literacy rate played a vital role in promoting communication channels to reach the right customers. Based on the above discussion, this study used the adult literacy rate (% of people ages 15 and above) as a correct variable for information sharing about coronavirus, and considers it as word-of-mouth (as denoted by WOM) information novel coronavirus among the general masses in this study. The rationale for using this proxy is that the literate person would effectively use all kinds of communication among agents. Hence, this proxy would leave the impact on the literature of network awareness.

(II) Lockdown: the lockdowns, either partial or complete, depend upon the severity of the new coronavirus outbreak in any country. This strategy used almost every country in their perspectives to prevent their ordinary peoples from the deadly disease. The evidence indicates that lockdown is not successful in many parts of the world due to the high incidence of poverty and hunger, which were later funded by the government’s emergency reliefs’ packages for the needy peoples [20]. The law enforcement agencies played an essential role in lockdown in the city, as per Federal government instructions [21]. The people did not usually follow the government instructions due to ignorance, a lack of information, and other social issues; for this call, law enforcement agencies can handle this situation. Thus, this study used ‘armed forces personnel’ (in total) for a nearby LOCKDOWN proxy to restrict free mobility. Measuring “lockdowns” by armed forces personnel is used to show a stringent government policy that, using ‘power and control’ to contain widespread coronavirus cases by the forceful imposition of standard operating procedures (SOPs) regarding coronavirus prevention, likely shows a better proposition than the ‘Oxford stringency index’ or ‘Google mobility series’.

(III) Social Distancing: according to the WHO guidelines regarding preventing and controlling the coronavirus pandemic, it avoids massive gatherings and maintains physical distancing among the residents. This strategy is mostly applied uniformly across the globe. Physical distancing helps to minimize the risk of coronavirus incidence as it is a transmitted disease, and its spread from close contacts [22]. The population compactness could be one reason that provides a channel to carry one person to another [18]. This study used ‘population density’, as per square km of land area, as a nearby proxy for the social distancing (denoted by SOCDIS) to obtain some conclusive findings in this regard. The study measures “social distancing” by population density, rather than using % of the urban population because, the higher the population compact in the country, the greater will be the chances to spread coronavirus cases, irrespective of rural and urban spheres. The study did not limit population density to the urban population while it used the overall population compactness, as coronavirus cases are spreading uniformly in rural and urban regions.

(IV) Price Control: due to the coronavirus outbreak, the globalized world’s most critical concern is the ‘price control’ of the food items especially. As the news about the COVID-19 outbreak transmitted across the globe, mass panic spread among ordinary people, and they rushed at food items to store in their homes. Every time, the governments give confidence to the familiar people and the producers and retailers to keep calm and remain easy so that food challenges can be resolved. In this regard, governments make food control price committees in a different part of the world to provide a free flow of food supply at lower prices [23]. The present study used the ‘consumer price index-inflation’ (%) as a proxy of food price control in the sense that the coronavirus pandemic increases the prices of food items due to the shortage of the food supply chain. Thus, the need to assess the price hikes can be used through CPI values for making an effective price control strategy.

(V) Financial and Economic Activities: the outbreak of novel coronavirus negatively affects the global stock market index. It is crushed in many parts of the world, due to full travel restrictions, lockdowns, and other preventive measures, which directly hit the local and international businesses [24]. The study used ‘broad money supply’, as % of GDP and ‘GDP per capita’ in constant 2010 US$ as nearby proxies of financial activities (denoted by FACT) and economic activities (EACT), respectively, to assess the country’s economic and financial situation amid the coronavirus pandemic. The rationale to use both of the factors is that money supply is considered to be one of the vital factors of financial development indicators that mostly viewed in the relation of COVID-19 pandemic. Similarly, economic activities can be better checked with the country’s per capita income that mainly affected the pandemic recession. Thus, these proxies would be helpful in tracing the real problem of the pandemic recession across countries.

(VI) Causes of Death by Communicable Diseases: the study used the data of ‘causes of death by communicable diseases’ (as % of total) (denoted by COMD) as a reference point to analyze the death toll by a coronavirus.

(VII) Services Value Added: the service’s value added (% of GDP) (as denoted by SVAD) comprises distributive services, producer services, personal services, and social services, which is used in this study as a response variable.

The COVID-19 measures are considered to be explanatory variables of the study, while the value of the service added is served as the explained variable. The world aggregated data are used for empirical analysis, covering a period of 1975–2020. The missing information is filled by the preceding and succeeding value of the respective variables where required. The data were obtained from the World Bank [25].

The study benefited from the Keynesian theory of aggregate demand, which argued that aggregate demand could be affected by any prevailing shocks in the economies, which need to be stabilized through economic policies. Similarly, the COVID-19 crisis is more severe than the financial depression of 2008 shocks that captured the whole world, which declined world economic growth. The COVID-19 crisis has affected the economies’ supply and demand simultaneously; for instance, social distancing creates distancing between the one person and another. It reduces productive labor hours that lower the supply, which increases the marginal cost of production [26,27]. Significantly, the services sector is majorly affected through social distancing, as its closely connected with the hospitality and recreational activities that are banned due to the high risk of spreading COVID-19 cases. Services value-added is a substantial part of economic growth, as its GDP share is more than 60% worldwide. The services sector is used as a reference point that analyzed its performance in the world’s GDP that is most affected by COVID-19 pandemic, which can be viewed by the suggested empirical equation, i.e.,

(1) $$\begin{array}{l} \ln(SVAD)=\alpha_{0}+\alpha_{1}\ln(COMD)+\alpha_{2}\ln(WOM)+\alpha_{3}\ln(LOCKDOWN)+\alpha_{4}\ln(FACT)+\alpha_{5}\ln(EACT)\\ +\alpha_{6}\ln(PCONT)+\alpha_{7}\ln(SOCDIS)+\varepsilon \\ ∴\frac{\partial \ln(SVAD)}{\partial \ln(COMD)}<0,\frac{\partial \ln(SVAD)}{\partial \ln(WOM)}>0,\frac{\partial \ln(SVAD)}{\partial \ln(LOCKDOWN)}<0,\frac{\partial \ln(SVAD)}{\partial \ln(FACT)}<0,\frac{\partial \ln(SVAD)}{\partial \ln(EACT)}<0,\\ \frac{\partial \ln(SVAD)}{\partial \ln(PCONT)}<0,\frac{\partial \ln(SVAD)}{\partial \ln(SOCDIS)}<0. \end{array}$$

where SVAD shows the services value-added, COMD shows communicable diseases, WOM shows word-of-mouth, LOCKDOWN shows lockdown, FACT shows financial activities, EACT shows economic activities, PCONT shows price control, SOCDIS shows social distancing, and *ε* shows the error term.

**Figure 1:**
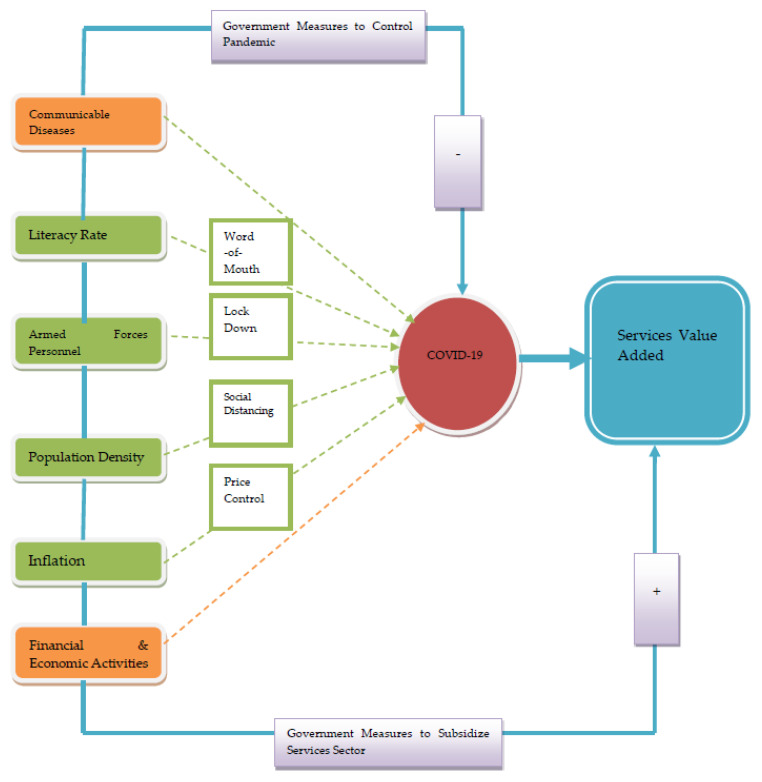
Research Framework of the Study. Source: Author’s extract.

Equation (1) shows that the stated factors influence service value-added. It is likely that the causes of death by communicable diseases, including COVID-19, will decrease service value-added, whereas improving the communication means of information sharing, including word-of-mouth about coronavirus pandemic, would be helpful in maintaining service value-added share relative to GDP. Although it is not favorable to the value of the functions added in managing their GDP share, the temporary or complete lockdown is not favorable. However, it is deemed to be desirable to control coronavirus on a global scale. The financial and economic activities suppressed with the COVID-19 pandemic negatively influenced services value-added. The price hikes in food items needed efficient price control to facilitate the needy community members, which subsidized the services sector to charge a smaller price, thus maintaining reasonable profit. Finally, social distancing is the remedial measure to contain coronavirus; however, it negatively affects service value-added. Figure 1 shows the research framework of the study.

Figure 1 shows the impacts of COVID-19 measures on the services industry and identified some significant determinants that negatively affect global service value added. These COVID-19 measures are highly required for the controlled pandemic; however, it decreases services share relative to its country GDP. The following research hypotheses have been developed to analyze it during estimation, i.e.,

**Hypothesis** **1** **(H1).** *Communicable diseases, including COVID-19, will likely decrease the share of services value-added relative to the country’s GDP*.

**Hypothesis** **2** **(H2).** *Word-of-mouth of coronavirus pandemic would likely to be helpful for the prevention of virus and increases services value-added, and*

**Hypothesis** **3** **(H3).** *Lockdown, population compactness, and financial instability will likely decrease services share in total GDP*.

The study utilized a quantile regression apparatus to obtain parameter estimates. It works under different assumptions. It gives a more trending analysis of the said parameters at different quantiles distribution, which other available regression apparatuses would be powerless to perform, such as time-series cointegration techniques, instrumental regression techniques, and robust regression. These techniques would perform well in their domain, but these are ineffective in analyzing trending regression estimates over 10th quantiles to 90th quantiles. The given procedure would give greater leverage to express the parameter estimates for sound inferences. Equation (2) shows the empirical illustration of different quantiles distribution of the stated parameters for ready reference, i.e.,

(2) $$\begin{array}{l} \ln{\left(SVAD\right)}_{\tau 10}=\alpha_{0}+\alpha_{1}\ln{\left(COMD\right)}_{\tau 10}+\alpha_{2}\ln{\left(WOM\right)}_{\tau 10}+\alpha_{3}\ln{\left(LOCKDOWN\right)}_{\tau 10}+\alpha_{4}\ln{\left(FACT\right)}_{\tau 10}\\ +\alpha_{5}\ln{\left(EACT\right)}_{\tau 10}+\alpha_{6}\ln{\left(PCONT\right)}_{\tau 10}+\alpha_{7}\ln{\left(SOCDIS\right)}_{\tau 10}+\varepsilon_{\tau 10}\\ ;\\ \ln{\left(SVAD\right)}_{\tau 25}=\alpha_{0}+\alpha_{1}\ln{\left(COMD\right)}_{\tau_{25}}+\alpha_{2}\ln{\left(WOM\right)}_{\tau 25}+\alpha_{3}\ln{\left(LOCKDOWN\right)}_{\tau 25}+\alpha_{4}\ln{\left(FACT\right)}_{\tau 25}\\ +\alpha_{5}\ln{\left(EACT\right)}_{\tau 25}+\alpha_{6}\ln{\left(PCONT\right)}_{\tau 25}+\alpha_{7}\ln{\left(SOCDIS\right)}_{\tau 25}+\varepsilon_{\tau 25}\\ :\\ \ln{\left(SVAD\right)}_{\tau 50}=\alpha_{0}+\alpha_{1}\ln{\left(COMD\right)}_{\tau 50}+\alpha_{2}\ln{\left(WOM\right)}_{\tau 50}+\alpha_{3}\ln{\left(LOCKDOWN\right)}_{\tau 50}+\alpha_{4}\ln{\left(FACT\right)}_{\tau 50}\\ +\alpha_{5}\ln{\left(EACT\right)}_{\tau 50}+\alpha_{6}\ln{\left(PCONT\right)}_{\tau 50}+\alpha_{7}\ln{\left(SOCDIS\right)}_{\tau 50}+\varepsilon_{\tau 50}\\ :\\ \ln{\left(SVAD\right)}_{\tau 75}=\alpha_{0}+\alpha_{1}\ln{\left(COMD\right)}_{\tau 75}+\alpha_{2}\ln{\left(WOM\right)}_{\tau 75}+\alpha_{3}\ln{\left(LOCKDOWN\right)}_{\tau 75}+\alpha_{4}\ln{\left(FACT\right)}_{\tau 75}\\ +\alpha_{5}\ln{\left(EACT\right)}_{\tau 75}+\alpha_{6}\ln{\left(PCONT\right)}_{\tau 75}+\alpha_{7}\ln{\left(SOCDIS\right)}_{\tau 75}+\varepsilon_{\tau 75}\\ :\\ \ln{\left(SVAD\right)}_{\tau 90}=\alpha_{0}+\alpha_{1}\ln{\left(COMD\right)}_{\tau 90}+\alpha_{2}\ln{\left(WOM\right)}_{\tau 90}+\alpha_{3}\ln{\left(LOCKDOWN\right)}_{\tau 90}+\alpha_{4}\ln{\left(FACT\right)}_{\tau 90}\\ +\alpha_{5}\ln{\left(EACT\right)}_{\tau 90}+\alpha_{6}\ln{\left(PCONT\right)}_{\tau 90}+\alpha_{7}\ln{\left(SOCDIS\right)}_{\tau 90}+\varepsilon_{\tau 90} \end{array}$$

where *τ*_10_ to *τ*_90_ show quantiles regression estimates from 10th quantiles to 90th quantile distribution.

The study further used impulse response function (IRF) and variance decomposition analysis (VDA) for analyzing the parameter estimates in the forecasting framework for the next ten year time period.

## 3. Results

Table 2 shows the descriptive statistics of the candidate variables. The share of service value that is added to world GDP has reached a maximum of 65.26%, minimum at 54.24%, and mean 58.47%. The causes of death by communicable diseases, on average, are entered at 27.29% of total world death. Word-of-mouth is measured by an adult literacy rate with a minimum value of 65.19%, a maximum amount of 87.30%, and an average value of 77.30%. Armed forces personnel are used as a proxy for lockdown, which shows that the global world needed 25,744,314 armed forces personnel to keep successful lockdown to some specified area on average. The financial and economic activities are measured by broad money supply and GDP per capita, with an average value of 92.75% of GDP and US$8,007.36, respectively. The price control is measured by changes in the price level with an average value of 6.39%. Finally, social distancing is observed by population compactness, which has an average value of 45.95 people per square m of land area. The given descriptions of the candidate variables showed a trend analysis over the past 45 years.

Table 3 shows the correlation estimates and found that communicable diseases, including COVID−19 and price control, negatively correlate with service value-added. In contrast, the other variables, including word-of-mouth, lockdown, financial and economic activities, and social distancing, positively associate services value-added. The result implies that services value-added exposed an increased risk of coronavirus pandemic, while strict price control further decreases services value-added across the globe. The government’s measures to controlled coronavirus would be primarily supported services value added to run their businesses during a relaxed time as per governments’ provision to open their markets. The word-of-mouth for coronavirus pandemic to the general masses would help keep residents at their homes to become safe from the virus, while, for successful operating lockdowns, the increasing number of armed forces personnel is desirable. Financial and economic activities allow general masses to start their businesses under strict government safety measures at their business site. Finally, social distancing is the only step that helps the broad population to keep away from the coronavirus; thus, avoiding massive gatherings and close contacts would enable peoples to do their work under the safety parameters. All of this positivity would support services value-added on a global scale.

Table 4 shows the ADF unit root estimates and found that, except SOCDIS, the remaining variables exhibit the first difference stationary, while SOCDIS does not show either I(0) or I(1) characteristics, thus it does not confirm the order of integration at the level or first difference. Based on the estimates, the study moves towards quantile regression estimates to show the variations of variables at different quantiles distribution.

Table 5 shows the quantile regression estimates and found that communicable diseases hurt the services value added at different quantiles distribution with a minimum impact of −0.068% and maximum impact of −0312%, while an increasing one per cent increase in services value-added share to the globe GDP. The results are interpreted in light of the novel coronavirus. Kim et al. [28] argued that community health is mainly influenced by the coronavirus outbreak that has increased the healthcare burden in national healthcare bills. The case study of New York city developed some protocols for the outpatient service department to minimize the risk of coronavirus pandemic, including a first stage, the possible test of coronavirus is performed on the susceptible patients. If found to be positive, then the second step is to give symptomatic treatments. The third stage is to track the patients once during at least five consecutive days, and, finally, teach them how to isolate in-home or elsewhere under prescribed medical guidelines. Samarathunga [29] discussed the possible challenges of the coronavirus pandemic on international tourism in Sri Lanka. The results show that the coronavirus pandemic negatively affects the country’s tourism sector, as it adversely affects the source markets, local tourism resources, and travel industry. The suspension of transportation modes, partial and complete lockdowns, and maintaining the distance between humans all decline tourism income. However, these measures are essential in containing coronavirus in a country. Yang et al. [30] concluded that, due to the high health risk of coronavirus pandemics to the national and international tourists, the tourism demand decreases through government institute bans on human mobility. Further, travel restrictions that are imposed by the government exacerbate adverse outcomes from the tourism sector. Thus, social welfare is the subject matter and prime responsibility of the government. Any strict policies regarding their prevention are desirable. However, the governments should subsidize the tourism sector to improve tourism sites; once the pandemic vanishes, an enormous amount of tourism revenue could be generated. Wanjala [31] argued that novel coronavirus negatively affects a country’s economic growth via low international trade and tourism transmission mechanisms. The travel and transportation restrictions for possible caution to take care of the humans from coronavirus are desirable, being substituted by the specific government-initiated reforms packages to the tourism and trade to maintain economic activities countrywide.

**Figure 2:**
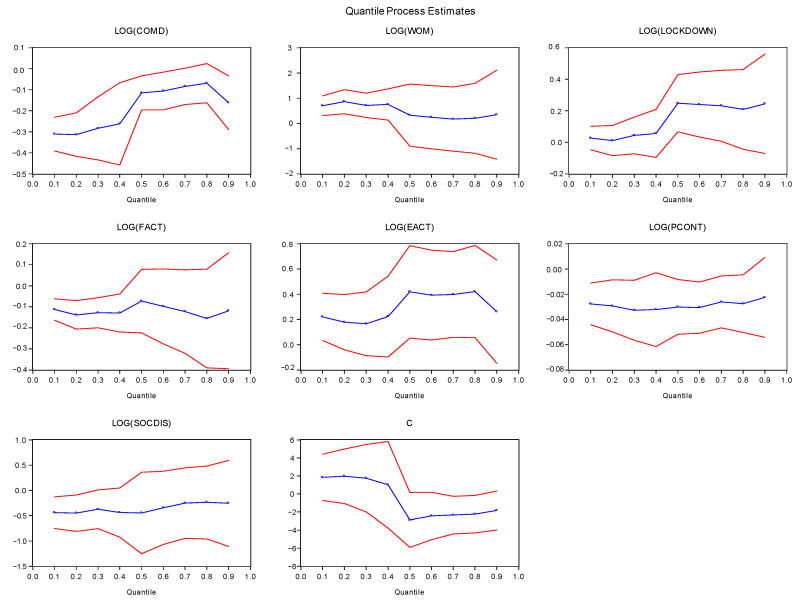
Quantile Process Estimates. Source: Authors’ estimates. Note: SVAD shows services value-added, COMD shows communicable disease, WOM shows word-of-mouth, LOCKDOWN shows lockdown, FACT shows financial activity, EACT shows economic activity, PCONT shows price control, and SOCIDIS shows social distancing. LOG shows natural logarithm. C shows constant. Red lines shows the critical region.Blue line shows the estimated value.

The sound financial activities, price control measures, and social distancing have proven to be the best strategy to control coronavirus; however, these measures negatively impact services value-added, leading to a global depression. The positive impact of word-of-mouth, lockdown, and sound economic activities decreases the risk of coronavirus pandemic and supports the services share into the world GDP. These results have been shown at different quantiles distribution. Brodeur et al. [32] discussed the vulnerability of the COVID-19 pandemic at a mass scale across the globe. The government put many efforts to restrain coronavirus through multiple strategies. However, the unified adopted policy included lockdown, which bears multifaceted mental health challenges to population well-being that are not limited to boredom, loneliness, sadness, worry, suicidal thoughts, stress, and divorce. The need for smart lockdowns and information sharing among the masses to stay safe in homes would be desirable, while the government should engage their population in some online group tasks to reduce mental health challenges. Wong [33] described the real situation of the coronavirus pandemic in the Malaysian context, where the physical distancing along with the national lockdowns were enforced with the one order command that local population from international travels, not allowing foreigners to visit a country, temporary shut down of businesses, closure of schools, colleges, and other institutions. At the same time, only essential services have been permitted under safety measures. These measures affect industries, including the services industry, which may cause a global depression. Barro et al. [34] found that the coronavirus pandemic and Spanish flu increase mortality and economic contraction, mostly low real returns on stocks and short-term government bills. Gómez-Ríos et al. [35] concluded that the coronavirus pandemic was mainly out of control due to the imported number of cases from uncontrolled air travellers. Social distancing avoids massive gatherings and restricts international travelling to maintain the decreasing trend in the infections trend. Yezli and Khan [36] argued that, besides the socio-economic, political, and religious challenges faced by the Kingdom of Saudi Arabia, the country took bold steps to restrain coronavirus through social distancing and complete lockdown. The country suddenly closed due to the high epidemic curve because of its social and religious norms hosting massive religious gatherings. These measures are essential in containing the virus, although at the cost of a severe economic crisis. The services industry mainly suffers due to restrictions being imposed on the travel and tourism sector, businesses shut down, closure of educational and other institutions, and maintaining social distancing; all of these measures would help to restrain the country’s epidemic curve. Figure 2 shows the quantile process estimates for ready reference.

## 4. Discussion

Table 6 shows the endogeneity test results that were performed through quantile median regression. Financial development generally works as a growth proxy; thus, evaluating the possible endogeneity in the given model, the study performs the three-step procedure. The first step is to use ln (FACT) as a dependent variable that is replaced by ln (SVAD), while the remaining variables are exogenous variables and obtained its residual value (i.e., res_01). In the second step, ln (SVAD) is used again as a primary endogenous variable, while res_01 and other variables, except for ln (FACT), are used as regressors and obtain coefficient estimates. In the final step, the Wald coefficient restrictions are applied on the given res_01 term and found the statistically insignificant results of t-statistics, F-statistics, and Chi-square statistics. The results confirmed that there are no possible endogeneity issues in the quantile regression estimates. Thus, the results are valid and reliable.

Table 7 shows the IRF estimates and suggested that smart lockdown services will positively influence services value-added, sound financial and economic activities, price control, and social distancing. In contrast, word-of-mouth and communicable diseases largely influenced services value-added that required a substantial information sharing system and increased healthcare expenditures to control the coronavirus pandemic and improve the share of services value added to the global GDP a time horizon.

Table 8 shows the VDA estimates and suggested that social distancing would exert a more significant magnitude to influence services value-added with a variance error shock of 43.3%, followed by economic activity, word-of-mouth, price control, communicable diseases, and financial business with variance errors of 24.6%, 11.1%,6.0%, 5.6%, and 0.302%, respectively. The least variance error shock of service value-added services will be lockdown with an estimated variance error shock of 0.09%.

## 5. Conclusions

The novel coronavirus is transmitted from one person to another through close contacts, coughing, sneezing, and touching. Hence, the governments adopted several policy instruments to contain coronavirus across the globe. The COVID-19 prevention measures are essential for breaking down the transmission channels, which would ultimately support the vanished coronavirus pandemic. In the recent crisis of the increased epidemic curve, the services sector is mainly affected due to lockdowns, travel, tourism, transport-related restrictions, business shutdown, and meagre economic and financial activities on a global scale. This study examined the possible impacts of COVID-19 measures on the services industry by using aggregated world data between 1975 and 2019. The results show that the causes of death by communicable diseases, including COVID-19, low financial activities, price control, and maintaining physical distancing, negatively affected the services value-added. In contrast, increased word-of-mouth regarding coronavirus pandemic in general masses, and smart lockdowns subsidized the services sector’s value across the globe. The study evaluated the COVID-19 pandemic’s impacts on the service industry in the inter-temporal forecasting relationship and suggested that smart lockdowns, sound economic policies, efficient price control mechanisms, and physical distancing will largely control the coronavirus, which leads to an increase in services share relative to the world GDP, over a time horizon. In this regard, the study proposed the following policy implications for a healthier contribution in the research community at a global scale, i.e.,

(I) There is a high need to control the possible transmission channels through which coronavirus is sustained for at least 14 days in the human body and found to uphold another carrier. It could mostly be contained through an improved information-sharing mechanism, which works as a word-of-mouth campaign through social media, print media, and other controlled information sources to spread knowledge regarding the deadly disease and suggest the ways to escape out from this pandemic. We have to think more strategically and look to new, innovative channels that are supposed to restrain coronavirus activities at a large scale, for instance, to promote subsidized nutritional supplements to the large population to obtain an increase in their immunity system and give emphasis to take healthy diets along with the supplements to produce resistance against the virus. Further, vigorous exercise workouts, brainstorming puzzles, indoor activities, and free internet and calling facilities may reduce the boredom and make them the excitement that encourages staying at home out of safety. On the other side, the governments offer some incentive packages to the whole-sellers, retailers, hotels, and business owners, so that they may be able to survive in a time of crisis. The lockdowns could be relaxed for some hours in a day and keep monitoring the business activities, so the risk of spreading coronavirus should be limited. The transportation and means of communications are mainly disturbed during this unprecedented time, affecting logistics activities and service quality. The governments should allow goods transportation at night time, which can freely reach the destination.

(II) Financial transactions and economic activities mainly suffered due to limited business opportunities in the crisis period. The nationwide lockdowns, social distancing, and inefficient price monitoring system led to more financial sector problems regarding loan disbursement and recovery. The small-scale industries have already been closed due to low demand, a sizeable number of people have been unemployed, and the related industries are shut down; all of these problems require smart solutions to manage the crisis period. The easy economic policies may attract investors to obtain a loan at less interest rate. However, the governments should have to create a demand and allow for some international goods movement in the form of exports and imports to channelize the financial activities.

(III) The community-based services should be initiated by involving local public administration, armed forces, healthcare workers, and academicians to provide fresh food-for-thoughts to get out from the misery. The public administration should build a liaison with the other stakeholders and business owners to schedule their work under the adoption of safety measures. A few suggestions can be beneficial in this regard, i.e.,

- all of the employees have to be regularly checked by the physicians and get health certification, so the workers may go inside the production unit and work accordingly;
- provide healthy, nutritious food in a meal to the employees to keep energy during the work process;
- maintaining the social distancing, as it is the root cause of spreading the pandemic;
- keep monitoring employees’ health and provide healthcare awareness to escape out from this pandemic; and,
- take care of their employees and motivate them through incentive-based work.

The armed forces can be effectively used to conduct lockdowns during the scheduled times. The healthcare workers played a decisive role in spreading mass awareness regarding methods of home isolation and their SOPs, while academics should have to teach students online at their homes and find a way to conduct assignments and quizzes, so the students get involved in their studies and obtain exam marks accordingly.

The COVID-19 measures to prevent the general masses from the deadly disease are the governments’ prime responsibility, while its impact should be regularly monitored to sustain economic activities in a country. The international tourism infrastructure is mainly damaged during the crisis period. It is a suggestion to make quarantine into tourists’ destination point, so the hotels and restaurants can utilize some subsidized payments, which helps to restore employment in this sector. The free flow of goods movement within and outside the country needed international SOPs and trading guidelines, so every country could get an equal opportunity. The smart lockdowns, accessible investment opportunities, charging low-interest rates, easy schedule of returning loans, food pricing control, maintaining physical distance in air-railways-road transportation, proper seating arrangement in keeping the gap between the seats and passengers, and tax rebates to the general public and industries could lessen the intensity of the coronavirus pandemic to support business-related activities across countries. The government should have to liberalize its economic policies to permit economic activities to maintain COVID-19 SOPs and monitor economic activities. Expansionary economic policies seem desirable for supporting vulnerable businesses. The second wave of coronavirus disease is exacerbated due to the failure of implementation in maintaining COVID-19 SOPs in highly dense cities and poor hygiene conditions, while the lack of a proper drainage system, garbage collection, and improper waste recycling system, as well of ignorance in adopting SOPs, make this pandemic more lethal. The COVID-19 vaccine would likely be available in the early or late 2021, although the priority is to infuse the vaccine to the healthcare physicians, staff, and older peoples, while, at a later stage, it will be available to the resident peoples. However, the government should adopt strict healthcare policies and COVID-19 SOPs to contain an enormous increase of COVID-19 cases for pandemic recovery. The study limited some suggested proxy factors that are closely linked to the government’s actions to contain the COVID-19 pandemic. Further research can be extended by using some real-time factors, including information and communication technologies, Oxford Stringency Index, Google Mobility series, and stock market indices, to assess pandemic recession in the globalized data set.

## Figures and Tables

**Table 1:** Assessment of Scholarly Writings on COVID-19 and Services Industry.

Words Operators	Searching Statistics	Words Operators	Searching Statistics	Words Operators	Searching Statistics
COVID-19 and Services Industry	No results found	COVID-19 and Hotel Business	No results found	Coronavirus and Defense	No results found
Coronavirus and Services Industry	No results found	COVID-19 and Restaurant Business	No results found	Coronavirus and Education	12 results
COVID-19 and Tourism	13 results	COVID-19 and Finance	2 results	Coronavirus and Health	134 results
COVID-19 and Transportation	8 results	COVID-19 and Entertainment	1 result	Coronavirus and Community Services	No results found
COVID-19 and Wholesale Business	No results found	COVID-19 and Recreational Activities	No results found	Total number of studies = 172	
COVID-19 and Retail Business	No results found	Coronavirus and Public Administration	2 results		

**Table 2:** Descriptive Statistics.

Methods	SVAD	COMD	WOM	LOCKDOWN	FACT	EACT	PCONT	SOCDIS
Mean	58.47710	27.29407	77.30327	25744314	92.75388	8007.362	6.394944	45.95990
Maximum	65.26177	30.90569	87.30101	30196640	125.0989	10892.00	12.47161	59.63624
Minimum	54.24299	20.17717	65.19396	22209230	62.15986	5681.743	1.431611	31.91508
Std. Dev.	4.384618	4.428943	7.039300	2934139	18.24449	1571.258	3.560119	8.506539
Skewness	0.212882	−0.521528	−0.341577	−0.087964	−0.073547	0.346740	0.464993	−0.013921
Kurtosis	1.326914	1.512059	1.738246	1.402185	2.251956	1.834101	1.970756	1.779506

Source: World Bank [25]. Note: SVAD shows services value-added, COMD shows communicable disease, WOM shows word-of-mouth, LOCKDOWN shows lockdown, FACT shows financial activity, EACT shows economic activity, PCONT shows price control, and SOCIDIS shows social distancing.

**Table 3:** Correlation Matrix.

Variables	SVAD	COMD	WOM	LOCKDOWN	FACT	EACT	PCONT	SOCDIS
SVAD	1							
	-----							
COMD	−0.925	1						
	(0.000)	-----						
WOM	0.912	−0.834	1					
	(0.000)	(0.000)	-----					
LOCKDOWN	0.726	−0.543	0.821	1				
	(0.000)	(0.000)	(0.000)	-----				
FACT	0.857	−0.809	0.953	0.708	1			
	(0.000)	(0.000)	(0.000)	(0.000)	-----			
EACT	0.946	−0.927	0.955	0.703	0.943	1		
	(0.000)	(0.000)	(0.000)	(0.000)	(0.000)	-----		
PCONT	−0.832	0.734	−0.894	−0.716	−0.887	−0.832	1	
	(0.000)	(0.000)	(0.000)	(0.000)	(0.000)	(0.000)	-----	
SOCDIS	0.931	−0.887	0.987	0.772	0.964	0.986	−0.875	1
	(0.000)	(0.000)	(0.000)	(0.000)	(0.000)	(0.000)	(0.000)	-----

Note: Small bracket shows probability value. SVAD shows services value-added, COMD shows communicable disease, WOM shows word-of-mouth, LOCKDOWN shows lockdown, FACT shows financial activity, EACT shows economic activity, PCONT shows price control, and SOCIDIS shows social distancing.

**Table 4:** Unit Root Estimates.

Variables	Level		First Difference	
	Constant	Constant with Trend	Constant	Constant with Trend
SVAD	−0.062 (0.947)	−2.152 (0.503)	−5.892 (0.000)	−5.879 (0.000)
COMD	−0.264 (0.921)	−2.196 (0.479)	−6.178 (0.000)	−6.741 (0.000)
WOM	−1.542 (0.503)	−0.631 (0.971)	−4.663 (0.000)	−4.922 (0.001)
LOCKDOWN	−1.617 (0.465)	−2.073 (0.545)	−7.663 (0.000)	−7.579 (0.000)
FACT	−0.386 (0.902)	−2.016 (0.576)	−5.932 (0.000)	−5.859 (0.000)
EACT	1.214(0.997)	−1.332 (0.866)	−5.028 (0.000)	−5.255 (0.000)
PCONT	−1.757 (0.396)	−3.107 (0.117)	−7.776 (0.000)	−7.718 (0.000)
SOCDIS	−0.972 (0.754)	−0.930 (0.943)	−0.730 (0.828)	−0.147 (0.992)

Note: small bracket shows probability values. SVAD shows services value-added, COMD shows communicable disease, WOM shows word-of-mouth, LOCKDOWN shows lockdown, FACT shows financial activity, EACT shows economic activity, PCONT shows price control, and SOCIDIS shows social distancing.

**Table 5:** Quantile Regression Estimates.

Quantiles	τ_10_	τ_20_	τ_30_	τ_40_	τ_50_	τ_60_	τ_70_	τ_80_	τ_90_
LOG(COMD)	−0.310	−0.313	−0.283	−0.262	−0.115	−0.105	−0.084	−0.068	−0.162
	(0)	(0)	(0)	(0.012)	(0.008)	(0.026)	(0.064)	(0.158)	(0.017)
LOG(WOM)	0.699	0.860	0.711	0.750	0.327	0.243	0.170	0.201	0.343
	(0.001)	(0.001)	(0.006)	(0.022)	(0.605)	(0.705)	(0.794)	(0.777)	(0.704)
LOG(LOCKDOWN)	0.027	0.010	0.043	0.056	0.247	0.239	0.231	0.208	0.243
	(0.484)	(0.823)	(0.470)	(0.472)	(0.011)	(0.028)	(0.051)	(0.114)	(0.137)
LOG(FACT)	−0.112	−0.138	−0.127	−0.129	−0.072	−0.097	−0.122	−0.155	−0.118
	(0)	(0)	(0.001)	(0.008)	(0.350)	(0.289)	(0.234)	(0.201)	(0.405)
LOG(EACT)	0.220	0.178	0.165	0.222	0.418	0.392	0.397	0.420	0.261
	(0.026)	(0.118)	(0.208)	(0.181)	(0.031)	(0.037)	(0.027)	(0.030)	(0.218)
LOG(PCONT)	−0.027	−0.029	−0.032	−0.032	−0.030	−0.030	−0.026	−0.027	−0.022
	(0.002)	(0.008)	(0.010)	(0.037)	(0.009)	(0.005)	(0.017)	(0.023)	(0.171)
LOG(SOCDIS)	−0.442	−0.451	−0.374	−0.439	−0.447	−0.346	−0.252	−0.239	−0.256
	(0.008)	(0.018)	(0.062)	(0.083)	(0.282)	(0.354)	(0.480)	(0.518)	(0.557)
Constant	1.834	1.959	1.741	1.020	−2.875	−2.435	−2.343	−2.239	−1.839
	(0.170)	(0.212)	(0.366)	(0.678)	(0.071)	(0.076)	(0.034)	(0.041)	(0.100)
Statistical Tests									
Slope Equality Test	Wald Test	χ^2^ –statistic: 37.055		χ^2^ –statistic degree of freedom = 14		Probability value: 0.000			
Symmetric Quantiles Test	Wald Test	χ^2^ –statistic: 13.616		χ^2^ –statistic degree of freedom = 8		Probability value: 0.092			

Note: Small bracket shows probability value. SVAD shows services value-added, COMD shows communicable disease, WOM shows word-of-mouth, LOCKDOWN shows lockdown, FACT shows financial activity, EACT shows economic activity, PCONT shows price control, and SOCIDIS shows social distancing.

**Table 6:** Endogeneity Test performed by Quantiles Median Regression.

*Variables*	First Step: ln (SVAD)				Second Step: ln (FACT)				Final Step: ln (SVAD)			
	*Coefficient*	*Std. Error*	*t-Statistic*	*Prob.*	*Coefficient*	*Std. Error*	*t-Statistic*	*Prob.*	*Coefficient*	*Std. Error*	*t-Statistic*	*Prob.*
*C*	−2.875	1.550	−1.854	0.071	6.848	4.894	1.399	0.169	−3.374	1.741	−1.937	0.064
*ln(FACT)*	−0.072	0.077	−0.946	0.350	N/A	N/A	N/A	N/A	N/A	N/A	N/A	N/A
*ln(COMD)*	−0.115	0.041	−2.789	0.008	0.434	0.175	2.469	0.018	−0.146	0.030	−4.818	0.000
*ln(WOM)*	0.327	0.628	0.520	0.605	−1.080	1.638	−0.659	0.513	0.406	0.679	0.598	0.553
*ln(lockdown)*	0.247	0.092	2.674	0.011	−0.355	0.218	−1.627	0.112	0.273	0.072	3.764	0.000
*ln(EACT)*	0.418	0.186	2.240	0.031	−0.005	0.538	−0.011	0.991	0.419	0.187	2.240	0.031
*ln(PCONT)*	−0.030	0.011	−2.717	0.009	−0.078	0.032	−2.427	0.020	−0.024	0.009	−2.477	0.017
*ln(SOCDIS)*	−0.447	0.410	−1.090	0.287	1.880	1.118	1.681	0.100	−0.584	0.480	−1.217	0.231
*Res_01*	N/A	N/A	N/A	N/A	N/A	N/A	N/A	N/A	−0.072	0.077	−0.946	0.350
Adjusted R^2^	0.852				0.779				0.852			
S.E. of regression	0.018				0.044				0.018			
Quantile dependent variable	4.080				4.537				4.080			
Sparsity	0.023				0.119				0.023			
Wald Coefficient Restrictions												
Wald Test	t-statistic: −0.946, *p* > 0.090				F-statistics: 0.896, *p* > 0.090				Chi-square statistic: 0.895, *p* > 0.090			

Note: SVAD shows services value-added, COMD shows communicable disease, WOM shows word-of-mouth, LOCKDOWN shows lockdown, FACT shows financial activity, EACT shows economic activity, PCONT shows price control, res_01 shows residual term, and SOCIDIS shows social distancing. N/A shows not applicable.

**Table 7:** Impulse Response Function (IRF) Estimates.

Response of SVAD								
Period	SVAD	COMD	WOM	LOCKDOWN	FACT	EACT	PCONT	SOCDIS
2020	0.487233	0	0	0	0	0	0	0
2021	0.190553	0.015831	0.083155	0.166405	0.048038	−0.142270	−0.020177	−0.128719
2022	0.046358	0.217748	0.246702	0.549362	−0.124709	−0.177730	−0.186485	−0.423154
2023	−0.281975	0.376039	0.442166	0.222861	−0.044556	−0.308266	−0.105952	−0.891468
2024	−0.984015	0.843134	1.169902	−0.028800	−0.137800	−1.591205	−0.521476	−2.367387
2025	−2.808627	2.151347	3.273988	−0.258479	−0.563898	−4.765879	−1.940319	−6.366720
2026	−7.404040	5.685884	8.494693	−0.770462	−1.497330	−12.39932	−5.735708	−16.45630
2027	−18.24873	14.34690	20.75606	−1.890141	−3.522563	−30.50206	−14.81691	−40.46431
2028	−43.56493	34.82272	49.22016	−4.514128	−8.166525	−72.90151	−36.00114	−96.68157
2029	−102.6897	82.82517	115.3919	−10.72818	−18.99123	−171.9555	−85.43064	−228.0026

Note: SVAD shows services value-added, COMD shows communicable disease, WOM shows word-of-mouth, LOCKDOWN shows lockdown, FACT shows financial activity, EACT shows economic activity, PCONT shows price control, and SOCIDIS shows social distancing.

**Table 8:** Variance Decomposition Analysis (VDA) Estimates.

Variance Decomposition of SVAD									
Period	SE	SVAD	COMD	WOM	LOCKDOWN	FACT	EACT	PCONT	SOCDIS
2020	0.487233	100.0000	0.000000	0.000000	0.000000	0.000000	0.000000	0.000000	0.000000
2021	0.589989	78.63170	0.072003	1.986502	7.955115	0.662960	5.814880	0.116955	4.759883
2022	1.010587	27.01061	4.667119	6.636374	32.26216	1.748787	5.074869	3.445038	19.15504
2023	1.546070	14.86678	7.909779	11.01467	15.86205	0.830233	6.143777	1.941547	41.43116
2024	3.723758	9.545757	6.490126	11.76918	2.740337	0.280061	19.31862	2.295818	47.56011
2025	10.22254	8.815312	5.290159	11.81905	0.427555	0.341449	24.29885	3.907338	45.10029
2026	26.90904	8.843002	5.228243	11.67120	0.143684	0.358904	24.73920	5.107267	43.90850
2027	67.05981	8.829139	5.418946	11.45926	0.102580	0.333716	24.67216	5.704278	43.47992
2028	161.4784	8.801253	5.585035	11.26718	0.095839	0.313321	24.63691	5.954317	43.34614
2029	382.2614	8.787153	5.691285	11.12292	0.095867	0.302734	24.63175	6.057192	43.31110

Note: SVAD shows services value-added, COMD shows communicable disease, WOM shows word-of-mouth, LOCKDOWN shows lockdown, FACT shows financial activity, EACT shows economic activity, PCONT shows price control, and SOCIDIS shows social distancing. SE shows standard error.

## Data Availability

The data is freely available at World Development Indicators published by World Bank [25] at https://databank.worldbank.org/source/world-development-indicators (accessed on 15 September 2020).
